# Lymph node tuberculosis mimicking axillary hidradenitis: a case report

**DOI:** 10.11604/pamj.2022.43.98.33903

**Published:** 2022-10-25

**Authors:** Zineb Basri, Ait Benhammou Rita, Amine Lakhdari, Maria Kharbouch, Hamza Tazi, Mounia El Omari

**Affiliations:** 1 Plastic and Reconstructive Surgery Unit, Cheikh Khalifa International University Hospital, Mohammed VI University of Health Sciences, Casablanca, Morocco

**Keywords:** Axillary hidradenitis, lymph node tuberculosis, axillary swelling, case report

## Abstract

Hidradenitis is a chronic benign pathology with acute manifestations, mainly occurring among young patients with hyper seborrhea. Its clinical presentation is an inflammatory, nodular and fistulized aspect. It affects many body parts, making its location a referral criterion for diagnosis. Our patient had, indeed, a clinical aspect of hidradenitis. However, pathology revealed that it is tuberculosis lymphadenitis.

## Introduction

Hidradenitis is a disease affecting the pilosebaceous follicle, associated with acne conglobata, pilonidal sinus, and scalp cellulitis, thus defining a tetrad. It affects 1 to 2% of the population, clearly predominately among females [1]. It most often occurs in the pubertal stage-rarely before puberty or menopause. Although it is a common condition, it remains unknown and is underdiagnosed at the first stage [2]. The diagnosis is essentially clinical with three well-established diagnostic criteria combined: a) “typical lesions made of painful nodules, abscesses, fistulas or hypertrophic scars; b) a specific topography-mainly of interest to folds; c) an evolution in chronic form, punctuated by flare-ups” [3]. We report the case of a young patient with a history of hyper seborrhea, displaying repeated episodes of unilateral axillary suppuration. According to our clinical examination, we considered it a hidradenitis. However, the histological examination will later reveal a tuberculous etiology. The patient background plus the location of the lesion is a typical presentation of hidradenitis. As far as we know, no similar case report makes the difference between these two diseases. The interest of our case is in a similar clinical presentation in addition to specific background data. The importance of proceeding to a pathology examination takes all its sense.

## Patient and observation

**Patient information**: the patient is 22 years old male with no medical history, non-smoking, obese with a BMI of 31 kg/m^2^ with no history of a tuberculosis contamination.

**Figure 1:**
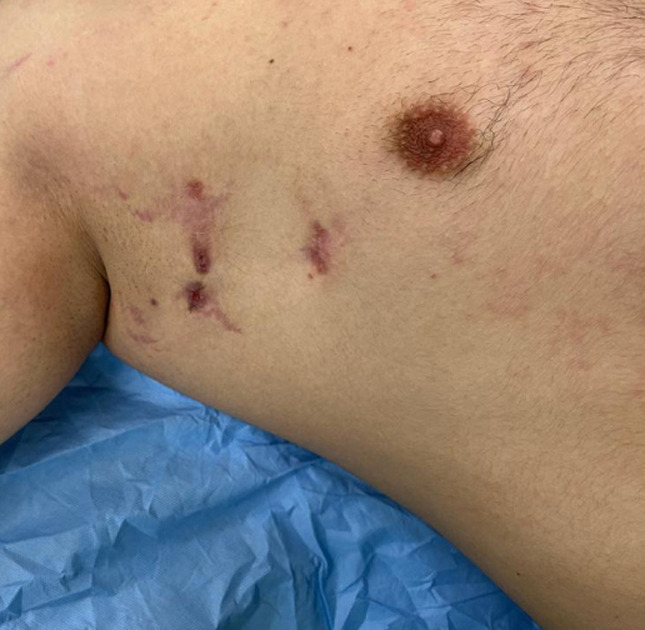
fistulas of the axillary area associated with lymphadenopathy

**Figure 2:**
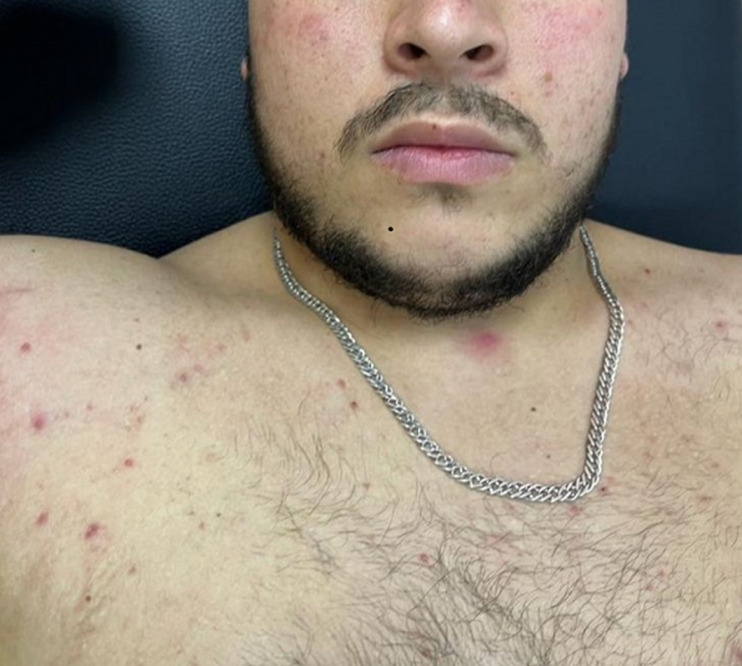
folliculitis located in the face and the chest area

**Clinical findings**: he consults for a right axillary suppuration evolving for two years with repeated episodes of abscessing, fistulization, and purulent flow. The initial lesion seemed to be an inflammatory induration of 2.5 cm, evolving by a push towards an extension on the surface and adherence to the deep plane. The evolution of the case was marked by periods of clinical improvement with the sagging of the renitent masses but then reappearing a few months later. This disabling situation altered the patient's quality of life and led him to many consultations during the last few years with protocols combining local care, antibiotics, and even repeated drainage of the collections. The examination at the admission finds an endure cupboard at the level of the right axillary region. It is 5cm in diameter with many fistulas and umbilicated papules adhering to the deep plane with ipsilateral lymphadenopathy (Figure 1). The rest of the skin examination spotlights lesions of predominant folliculitis at the back, the trunk, and acne on the face (Figure 2). Besides, the patient does not display any general infectious signs (no fever or chills, or night sweats).

**Figure 3:**
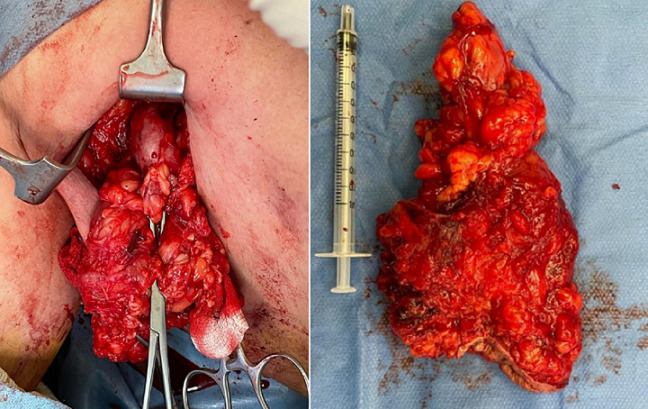
surgical specimen: skin flap carrying all the fistulas and its deep extension with profound adhering lymphadenopathy, visible on the image

**Therapeutic interventions**: we have opted for surgical exploration for diagnostic and therapeutic purposes. A wide surgical excision was performed in one piece, carrying the entire fistulized skin flap to the surface with its deep extensions and adhering lymphadenopathy to the magma of the inflammatory tissue (Figure 3). We maintained post-surgery drain care for 48 hours, a probabilistic antibiotic therapy approach, and local care until healing. The specimen has been oriented and sent for pathological examination.

**Figure 4:**
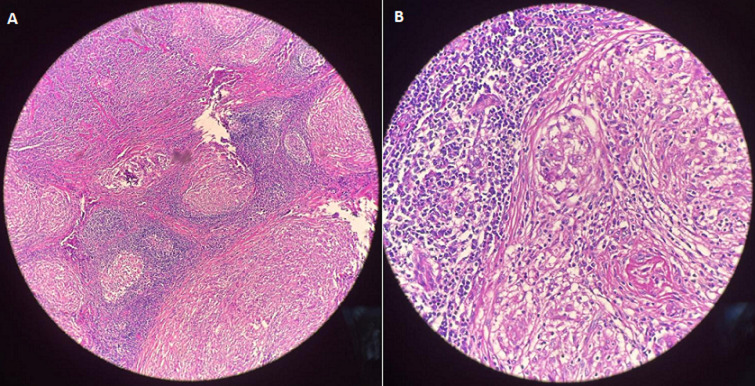
histological section A) granulomatosis lymphadenitis (HE, Gx40); B) same section (HE, GEx100)

**Diagnostic assessment**: histological analysis reveals the following: inflammatory lymphadenitis with caseous necrosis. Absence of signs of malignancy (Figure 4). The patient received treatment based on anti-tuberculosis therapy.

**Figure 5:**
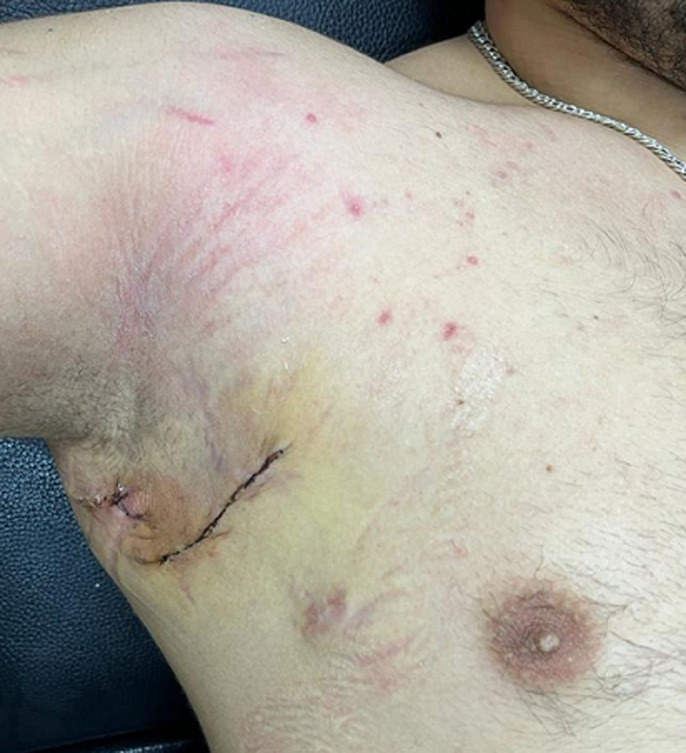
surgery outcome: intradermal suture postoperative

**Follow-up and outcome of interventions**: the postoperative evolution was good, with complete healing after a week and no recurrence (Figure 5).

**Patient perspective**: the patient was satisfied with the treatment and the postoperative results.

**Informed consent**: the patient provided full consent after an oral explanation of our intention of publishing her case.

## Discussion

Hidradenitis remains a condition that occurs mainly in women more than men, after puberty, with risk factors such as smoking and being overweight [4]. Its seat predominates at the level of the gluteal region in men and the submammary or axillary fold in women [3]. The elemental lesion is first nodular, abscessed, then fistulized, localized, or diffuse. Depending on the location, a multitude of differential diagnoses can be confusing. In front of a nodular axillary suppuration, the differential diagnoses are recurrent abscess, scrofuloderma (and, by extension, lymph node tuberculosis), and finally, inflammatory squamous cell cyst. On the one hand, affecting the pilosebaceous follicle, hidradenitis can lead to the inflammation and fibrosis of neighboring tissues with reactive lymphadenopathy and possible super-infection. On the other hand, lymph node tuberculosis initially occurs in axillary lymphadenopathy, progressing to fistulization of the skin. Super-infection and secondary fibrosis is this way possible [5]. Here is the importance of finding which symptom appears first, either the lymphadenopathy or the skin lesion.

In each situation, suppuration and inflammation phenomena go from one sense to another, either from the skin to reactive adenopathy (hidradenitis) or from the adenopathy to the skin (lymph node tuberculosis). The transition to chronicity can add further ambiguity, whether it is due to delayed diagnosis or therapeutic wanderings. The final clinical presentation is, therefore, the same. We can make a difference through the context and clinical data. A physician can quickly point to axillary hidradenitis for our patient: a chronic axillary suppuration with a background of hyper seborrhea and diffuse follicular lesions at the trunk and face. The endemic context in our country should also make us think of lesions of cutaneous tuberculosis in its form of scrofuloderma. This confusion leads us to conduct a precise interrogation and a complete clinical examination for signs that can point to an etiology underlying this ambivalent clinical presentation.

## Conclusion

Facing a chronic axillary suppuration, we should consider the diagnosis of hidradenitis. However, lymph node tuberculosis should not be ignored, especially in endemic countries. Consequently, we can reconsider pathology indications in front of axillary suppuration.
